# First Direct Evidence of Long-distance Seasonal Movements and Hibernation in a Migratory Bat

**DOI:** 10.1038/srep34585

**Published:** 2016-10-04

**Authors:** Theodore J. Weller, Kevin T. Castle, Felix Liechti, Cris D. Hein, Michael R. Schirmacher, Paul M. Cryan

**Affiliations:** 1 USDA Forest Service, Pacific Southwest Research Station, 1700 Bayview Drive, Arcata, California, 95521, USA; 2 Wildlife Veterinary Consulting, 840 Sundance Drive, Livermore, Colorado, 80536, USA; 3 Swiss Ornithological Institute, Seerose 1, 6204 Sempach, Switzerland; 4 Bat Conservation International, PO Box 162603, Austin, Texas, 78716, USA; 5 U.S. Geological Survey Fort Collins Science Center, 2150 Centre Avenue, Building C, Fort Collins, Colorado, 80526, USA

## Abstract

Understanding of migration in small bats has been constrained by limitations of techniques that were labor-intensive, provided coarse levels of resolution, or were limited to population-level inferences. Knowledge of movements and behaviors of individual bats have been unknowable because of limitations in size of tracking devices and methods to attach them for long periods. We used sutures to attach miniature global positioning system (GPS) tags and data loggers that recorded light levels, activity, and temperature to male hoary bats (*Lasiurus cinereus*). Results from recovered GPS tags illustrated profound differences among movement patterns by individuals, including one that completed a >1000 km round-trip journey during October 2014. Data loggers allowed us to record sub-hourly patterns of activity and torpor use, in one case over a period of 224 days that spanned an entire winter. In this latter bat, we documented 5 torpor bouts that lasted ≥16 days and a flightless period that lasted 40 nights. These first uses of miniature tags on small bats allowed us to discover that male hoary bats can make multi-directional movements during the migratory season and sometimes hibernate for an entire winter.

Individuals of several species of North American bats make biannual migratory journeys between winter and summer habitat^1^, yet compared to birds, our understanding of the details and destinations is nascent. In relative terms, we have extensive knowledge of bird migration that stems from the ease with which humans can observe seasonal changes in species occurrence and obvious group movements over continental scales, as well as the ability of larger animals to carry tracking devices^2^,^3^,^4^. Studying migration in the smallest flying animals remains a challenge. Long-distance movements of small (<30 g) birds were initially revealed through extensive banding (ringing) efforts^2^, and later by incorporating isotope analyses^5^. Recently, breakthroughs concerning the seasonal whereabouts, flight paths, and long-term activity patterns of small migratory birds have also been made possible by miniature global-positioning-system (GPS) tags^6^ and data-recording environmental sensors (hereafter data loggers^7^,^8^,^9^). Recent increases in our understanding of bird-migration discoveries were made using miniaturized (1–2 g) tracking and sensor devices^10^,^11^,^12^, which augurs well for advancing understanding of migration and seasonal behaviors in a particularly difficult-to-study group of long-distance migrants—small bats. As in birds^13^, there is growing recognition that effective conservation of bats requires understanding of their needs beyond the summer breeding season and winter hibernation periods when they are easiest to study^14^.

It has long been known that certain species of bats migrate^15^,^16^, but studying bats is extremely challenging because of their ubiquitously cryptic nocturnal activity patterns and secretive roosting. These difficulties have left broad gaps in our understanding of many bats, but particularly the small-bodied, long-distance migrants. Efforts to study bat migration have employed methods such as banding individuals^17^,^18^,^19^, visual observations and captures^20^,^21^, compiling seasonal maps of occurrence records^22^,^23^, genetic analyses^24^,^25^, radiotracking^26^,^27^, and stable isotope analyses^28^,^29^,^30^,^31^,^32^. However, the success rates and spatial or temporal resolution of such methods remain low. Although new technology recently enabled following the long-distance movements of large (>100 g) bats^33^,^34^, data on the movement patterns and behaviors of small migratory bats do not exist. Yet, as with birds^3^,^6^, knowledge of movements and behaviors of individual animals can provide important insights into their ecology and ultimately help provide for their conservation.

In North America, so-called “migratory tree bats” (sensu^35^) are thought to undertake some of the longest seasonal movements of any bat species. Hoary bats (*Lasiurus cinereus*) roost individually in the foliage of trees at low density and, despite a wider distributional range than most mammals, are rarely encountered through vast areas of their range^36^; these characteristics combine to make them one of the most poorly understood migratory tree bats. Seasonal distribution patterns inferred from occurrence records and stable isotope analyses indicate that hoary bats generally migrate southward and towards coasts from their summer range to overwinter^22^,^23^,^28^,^29^,^37^. Migration is often defined as seasonally mediated, directional movements between habitats^2^. Hence, although it can be expected that animals select migratory routes that minimize energetic costs, the precise movements of individual hoary bats were unknown. Conventional understanding has been that hoary bats move to areas of moderate climate for the winter which allows them to make frequent use of daily torpor interspersed with occasional feeding when insects are active^38^. However, evidence of extensive cold-season activity by hoary bats in any of their potential wintering areas is lacking.

Here, we describe our successful use of two types of miniature data-recording devices that allowed us to gain new insights into the ecology and behavior of individual hoary bats. We sutured GPS tags to male hoary bats and obtained multiple site locations that allowed us to infer long-distance movements of individuals during the autumn migration period. To other bats, we attached data loggers that recorded light level, temperature, and activity, from which we obtained detailed information on individual hoary bats over periods spanning as long as an entire winter. These data recorded from small, free-ranging, migratory tree bats are the first of their kind and allow us to challenge two assumptions about hoary bats: (1) that their autumn migration routes are directional and generally linear and (2) that, unlike smaller cave-dwelling bats, they do not hibernate or use sequential bouts of multi-day torpor during winter.

## Results

### Autumn Movements

**Figure 1:**
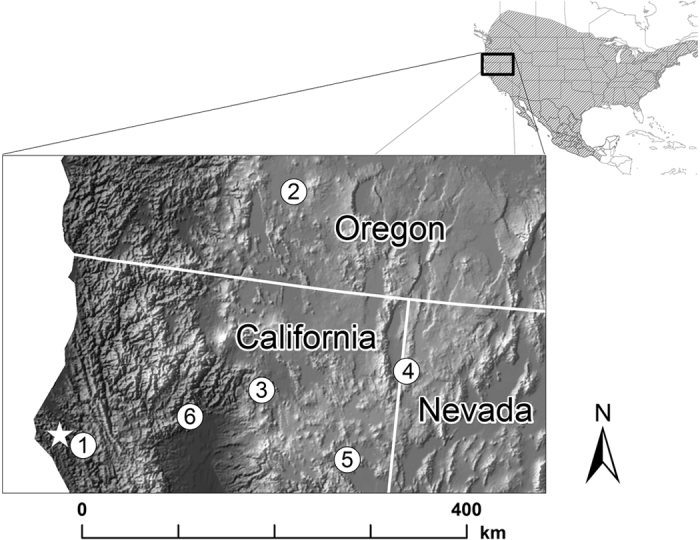
Locations of a free-ranging hoary bat (*Lasiurus cinereus*) recorded using a miniature GPS tag in October 2014. General location of sites where the male bat was captured and fit with GPS tag in September 2014 and then recaptured in April 2015 are illustrated with white star. Dates of bat locations (white circles) were: (1) Oct 1, (2) Oct 5, (3) Oct 12, (4) Oct 21, (5) Oct 25, and (6) Oct 28 2014. Inset map shows distribution of hoary bats in North America (hatched area) and region of detail (black rectangle). Map created using ArcGIS version number 10.3.1 (http://www.esri.com/software/arcgis).

We attached GPS tags to 8 male hoary bats in late September 2014 and recovered 3 of them after they had recorded GPS locations (hereafter ‘fixes’). In total, we obtained 2, 4, and 6 GPS fixes per bat that were recorded during October 2014. GPS data revealed 3 different behaviors of the bats we tracked: site fidelity, local (<100 km) movements, and long-distance (>100 km) movements. We recaptured Bat 479 on 4 different nights and obtained 4 GPS fixes from it over a period of 26 days. The longest movement recorded for Bat 479 was 6.4 km between its first GPS fix and its first recapture location. We recaptured Bat 481 twice following tag attachment leading us to document movements of 51.4 km and then a further 16.4 km southeast of our study area. The recapture of Bat 481 on Oct 5 2014 revealed it had traveled 67.8 km from its last GPS fix, recorded 25 hours earlier. We tagged Bat VHF5 on Sep 27 2014 and recaptured it on Apr 30 2015, 213 days after tag attachment. The resulting 6 GPS fixes, document that Bat VHF5 flew >1000 km during October 2014 in a large circuit that began and ended in the vicinity of the capture area (Fig. 1).

### Activity Patterns and Torpor Use

**Figure 2:**
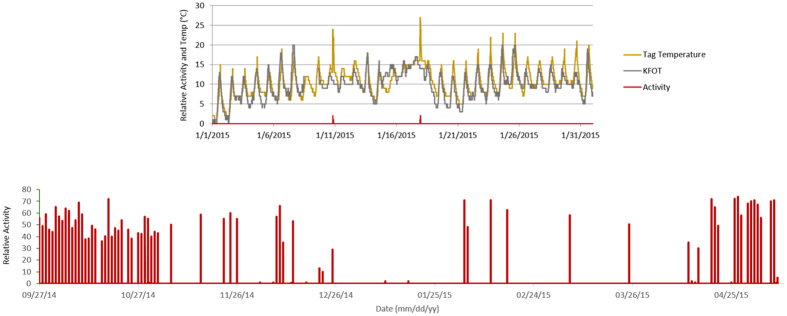
Activity of a male hoary bat during autumn 2014 – spring 2015 in relation to local environmental conditions. Relative activity levels (red lines) <6 are not associated with flight. Exploded view above shows tag temperature during January 2015 (yellow line), which tracked ambient temperatures at a nearby weather station (KFOT; gray line) except during 2 brief periods of rewarming by the bat without flight.

**Figure 3:**
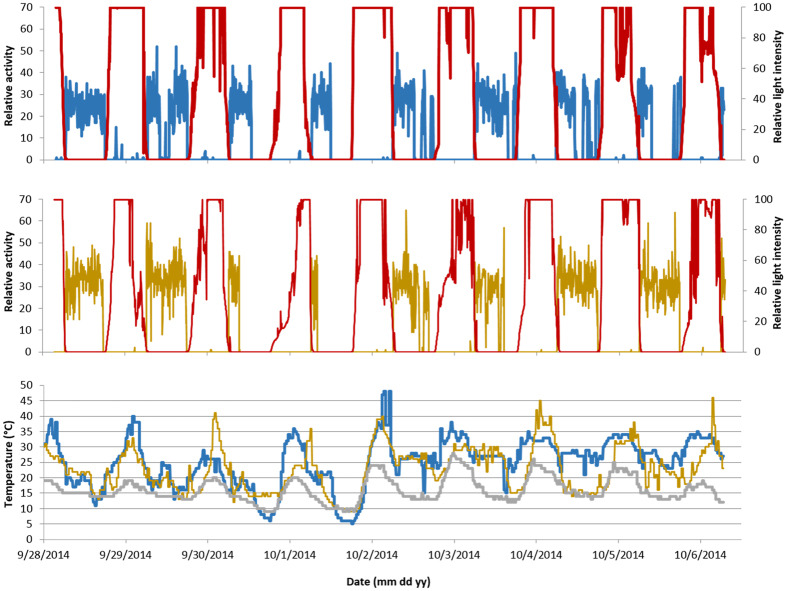
Comparison between activity patterns by two male hoary bats in northern California during autumn 2014. Relative light levels recorded by data loggers are depicted in red, ambient temperature from KFOT weather station in grey. Relative activity levels for blue-tagged bat (top panel), yellow tagged bat (middle panel), and tag temperatures for both bats (bottom panel). Date labels are centered on midnight.

We attached data loggers to 6 male hoary bats on Sep 26 and 27 2014. We recovered 1 of them that had recorded 9 days of data during autumn 2014 and another that had recorded 224 days of data from autumn 2014 through spring 2015 (Fig. 2). We obtained simultaneous activity data from both bats from Sep 27 to Oct 6 2014 (Fig. 3). While both bats were mostly active throughout the entire night, on Sep 30 and Oct 1 both were only active during the first half of the night. Ambient temperatures on both of these nights reached as low as 9 °C, whereas they remained ≥12 °C on other nights during this period (Fig. 3). Both bats entered torpor on the evening of Oct 1 as evidenced by cessation of activity and tag temperatures conforming to ambient temperatures. Differences in temperature sensor readings between the bats carrying data loggers were sometimes noted. We speculate that differences in tag temperature sensor readings between bats carrying data loggers could be associated with them occupying different areas. For example, on the evening of Oct 4, when the blue-tagged bat was active and presumably flying in an area that was about 10 °C warmer than the area where the yellow-tagged bat was active (Fig. 3).

**Figure 4:**
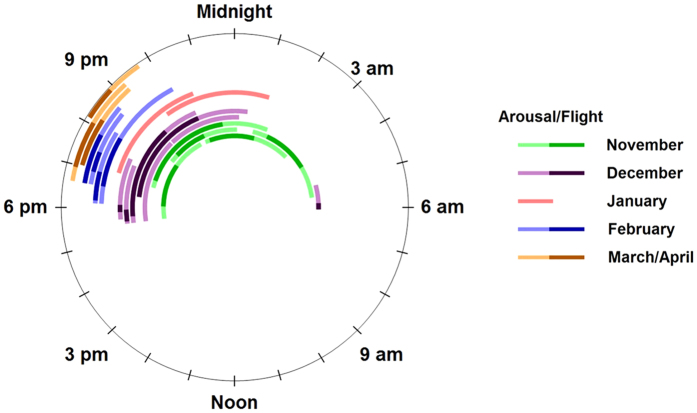
Arousal and flight events by a male hoary bat from 6 Nov. 2014–12 April 2015. Each arc extends between the beginning and end of an arousal from torpor. Darker shades represent the time period in which the bat was active, likely in flight, and lighter shades represent periods without flight activity, but when the tag was at arousal temperatures before and after a flight event. On 4 occasions, 2 in December and 2 in January, the bat aroused without exhibiting activity levels associated with flight.

We obtained a near-continuous record of activity, light exposure, and tag temperatures of a male hoary bat over 224 days from autumn through spring. Activity occurred on 31 of 34 nights in September and October, but then declined sharply in early November and did not increase substantially until late April (Fig. 2, Table 1). The bat was inactive on most nights during winter. Between Nov 3 2014 and Apr 12 2015, the bat exhibited levels of activity associated with flight on only 16 (10%) of the 160 nights monitored. The longest period of inactivity lasted 40 nights, from Dec 26 2014 to Feb 03 2015. We recorded 19-, 18-, and 18-night periods of inactivity separated by single nights of activity between Feb 16 and Apr 12 2015. When the bat was active between December 2014 and March 2015, it was generally active for <10% of each night (Table 1). During the period from Feb 03 to Apr 12 2015 the bat began flight activity an average of 53 minutes (range: 37–77 minutes) after sunset and was active for an average of 54 minutes (range: 30–80 minutes) (Fig. 4).

By comparing tag temperatures to ambient temperatures at weather stations in the region (Figs S1 and 2) we inferred that the yellow-tagged bat likely overwintered in the vicinity of where we captured it. Excluding two arousals that were not associated with flight, mean temperature of the tag during the 40-day inactive period that included January was 10.1 °C (range = 1–23). On average, the tag was 0.9 °C (range = −7.0–9.0) warmer than air temperatures at KFOT station (25 km NNW of capture area) and there was strong temporal correlation between the two temperatures (r = 0.88, Fig. 2) indicating that the bat was in torpor during this time. Similarly the bat remained in torpor during an 18-day period in mid-March despite the mean temperature of the tag reaching 14.3 °C (range = 8–27). We recorded 20 arousals from torpor by the bat between Nov 02 2014 and Apr 12 2015 (Fig. 4), 4 of which were not associated with flight. Arousals were more frequent and generally longer in duration during November and December than during January–April when they occurred, on average, 12 days apart. The mean duration of arousals over the entire winter was 199 (range 55–385) minutes.

## Discussion

We used two new types of technology to assess the ecology and behavior of hoary bats during migration and over-wintering. GPS tags allowed us to determine that some individuals make long distance, multi-directional movements during autumn while data loggers allowed us to demonstrate that hoary bats can engage in winter-long hibernation.

The three male hoary bats we followed exhibited a variety of movement behaviors during autumn. For one bat we had no evidence that it vacated the general vicinity of where it was captured, whereas another bat flew at least 68 km straight line distance in single night and a third completed a >1000-km circumnavigation of northern California, Oregon, and Nevada over the course of a month. Hence our results demonstrate the possibility that some individuals may not engage in relatively simple, directional movements during autumn. The reason for long-distance, round trip travel exhibited by the male hoary bats in our study is enigmatic. It is possible that the long-distance movements we documented were associated with bats seeking favorable conditions of temperature and humidity for roosting and foraging^39^. Although this explanation may account for movements to and from areas dozens of km away, it does not seem sufficient, energetically, to explain movements of >300 km from the study area. Another hypothesis, based on synchrony between autumn migration and mating readiness in hoary bats^40^, is that the male bats we tracked were trying to intercept and mate with females migrating to wintering grounds.

Hoary bats are inarguably a migratory species, yet we have shown with a single individual that they are capable of hibernating for a period of 6 months during winter. Although early laboratory research indicated that species of *Lasiurus* may be well-adapted for hibernation^41^, subsequent observations of free-ranging hoary bats using radio-telemetry had only documented multi-day bouts of torpor during summer^42^ and periods lasting less than one month during winter in eastern red bats (*Lasiurus borealis*)^43^,^44^. The number and length of torpor bouts and frequency of arousal we observed in a male hoary bat, particularly in late-winter, was generally similar to what has been observed for bats hibernating in caves and mines^45^,^46^,^47^. In fact, the bat we monitored remained in hibernation despite ambient temperatures at which it was active in the study area during autumn and which insect prey was likely available. Furthermore, the bat appeared to retain its circadian rhythm, because its arousals coincided with sunset (Fig. 4). Maintenance of a dusk-arousal circadian rhythm throughout winter has also been documented in cave-hibernating bats that live in regions with mild winters, whereas hibernating cave bats in regions with harsher winters tend to lose dusk-arousal rhythms during mid-winter^45^,^48^,^49^. Further, on 4 occasions the bat re-warmed without taking flight demonstrating that, as in other hibernating bats, arousals can be motivated by needs other than feeding^49^. These observations support the suggestion that hibernation is a conserved trait in temperate-zone bat species^50^. Knowledge that hoary bats can move long distances in non-linear ways and hibernate during winter may have practical impacts. For example, hoary bats frequently collide with wind turbines during autumn^51^ and are currently presumed to be safe from white-nose syndrome, an emerging disease that heretofore has only impacted cave-hibernating bats^52^. Continued use and enhancement of the tracking technologies we demonstrated on hoary bats could help advance understanding of bat biology, as well as some of the most important conservation issues currently involving bats.

## Methods

### Tag Attachment

We attached two types of tags to bats: programmable GPS tags with and without VHF transmitters (Pinpoint 8, Lotek Wireless, Newmarket, Ontario, Canada), and data logger tags (GDL3, Swiss Ornithological Institute, Sempach, Switzerland). The GPS-only tags weighed 1.1 g and had dimensions of 22.0 mm × 11.0 mm × 4.5 mm with a posterior-extending antenna 43 mm in length. The GPS tags were programmed to record location on 8 specified dates and times. Five GPS tags also included VHF transmitters (PicoPip AG317, Lotek Wireless, Newmarket, Ontario, Canada). Those tags weighed 1.4 g, and had dimensions of 20.5 mm × 15.0 mm × 6.0 mm with an additional posterior-extending antenna 145 mm in length. VHF transmitters were intended to help establish which animals were still in the study area during the first month after attachment. Both types of GPS tags were re-chargeable and re-programmable without removing the tag from the bat. We programmed GPS tags to record nighttime locations approximately 1 hour after local sunset, and daytime locations at noon. Data from GPS tags consisted of date and time of position, an estimate of tag location in 3 dimensions, PDOP a measure of location accuracy, and the time required to acquire position information. We estimated GPS location precision as approximately ±200 m based on tests conducted at ground level using tags that were both stationary and in motion.

We also attached 1.14 g data loggers with dimensions of 24.0 mm × 10.0 mm × 4.0 mm to hoary bats. The tags included a 5 mm long light sensor that extended dorso-caudally. Data loggers recorded light-level and animal activity via accelerometer, every 5 minutes similar to those used by Liechti *et al.*^11^; both values are relative, dimensionless, values. Temperature (°C) was measured at the dorsal surface of the tag every 30 minutes and was a mixture of ambient temperature and bat body temperature. Although temperature readings did not allow us to precisely determine bat body temperature, we were able to determine when hoary bats were euthermic either by comparison of tag temperature data to patterns in ambient temperature measured by weather stations (Fig. 2, Supplementary Information) and/or corresponding patterns in tag activity data indicating bat movement (Fig. 3). Tag temperature approximated ambient temperature when a bat was inactive and thermoconforming and increased above ambient when the bat was inactive but euthermic (Fig. S3) and when the tag was in direct sunlight.

We attached both types of tags to bats from Sep 22–27 2014. We captured hoary bats in mist nets along the channel of Bull Creek in Humboldt Redwoods State Park, California (latitude: 40.35, longitude: −124.01). Bats were captured in standard 2.6-m high mist nets and in a triple-high configuration with three standard mist nets stacked on top of one another. We attached tags to adult male hoary bats, which comprise >95% of captures at this site, selecting individuals with the highest mass captured on a given night. We attached tags to the dorsum, caudal to the scapulae and cranial to the pelvis using sutures following the methods of Castle *et al.*^53^. Bats were released at the capture site after allowing them 20 minutes to recover from anesthesia. Bats to which tags were attached did not exhibit unusual levels of mass loss, skin irritation, or mobility while entering roosts^53^.

We attempted to recapture tagged individuals using mist net surveys along the Bull Creek waterway on 19 nights between Sep 26 and Oct 19 2014, 13 nights between Oct 27 2014 and Apr 02 2015, and 22 nights between Apr 12 and 28 May 28 2015. When bats carrying GPS tags were recaptured during autumn 2014 we downloaded data and recharged and reprogrammed tags while they were attached to bats^53^. In contrast, recovery of data from data loggers required removal of the tag.

Bat capture and handling were carried out in accordance with guidelines of American Society of Mammalogists^54^ under permit with the California Department of Fish and Wildlife (#SC-002911). Our experimental methods were approved by the Institutional Animal Care and Use Committee of the U.S. Geological Survey Fort Collins Science Center (FORT IACUC 2014-08).

### Data Analysis

For data analysis we considered bats to be active when 5-min activity values exceeded a relative activity level of 6 (on a scale of 0–74), based on a comparison with activity levels logged during daylight hours when the bats were roosting. We considered the nighttime period to be the 5-minute observations between sunset and sunrise in our study area, although this will be inaccurate if bats moved >100 km from our study area. We considered the bat to be torpid when it was inactive and within 4 °C of the temperature at a nearby weather station (Figs S1 and 2). We defined an arousal as a total increase of ≥3 °C in tag temperature that occurred at night in the absence of similar increase in ambient temperatures at nearby weather stations.

## Additional Information

**How to cite this article**: Weller, T. J. *et al.* First Direct Evidence of Long-distance Seasonal Movements and Hibernation in a Migratory Bat. *Sci. Rep.* **6**, 34585; doi: 10.1038/srep34585 (2016).

## Supplementary Material

Supplementary Information

## Figures and Tables

**Table 1:** Number of nights on which a male hoary bat (yellow-tagged) exhibited activity from 28 Sept 2014–8 May 2015, as recorded by a data logger on the bat’s back.

Month	Nights Tagged	Active Nights	Percent Nights Active	Mean (range) Proportion of Night Active*
September	3	3	100.0	0.705 (0.251–0.938)
October	31	28	90.3	0.443 (0.013–0.959)
November	30	7	23.3	0.293 (0.097–0.847)
December	31	5	16.1	0.095 (0.005–0.227)
January	31	0	0.0	0.000
February	28	4	14.3	0.074 (0.037–0.103)
March	31	2	6.4	0.097 (0.094–0.101)
April	30	9	30.0	0.302 (0.039–0.626)
May	8	6	75.0	0.359 (0.067–0.517)

^*^Mean proportion of 5-minute periods during the night that the bat was active were calculated only for nights when bat was active.
