# Supplementary material for: First Direct Evidence of Long-distance Seasonal Movements and Hibernation in a Migratory Bat
Source: Sci Rep. 2016 Oct 4;6:34585. doi: 10.1038/srep34585 (PMC5048302; doi:10.1038/srep34585)
Supplement: Supplementary Information [file srep34585-s1.pdf]

SUPPLEMENTARY INFORMATION

First Direct Evidence of Long-distance Seasonal Movements and Hibernation in a Migratory Bat

Theodore J. Weller, Kevin T. Castle, Felix Liechti, Cris D. Hein, Michael R. Schirmacher, Paul M. Cryan

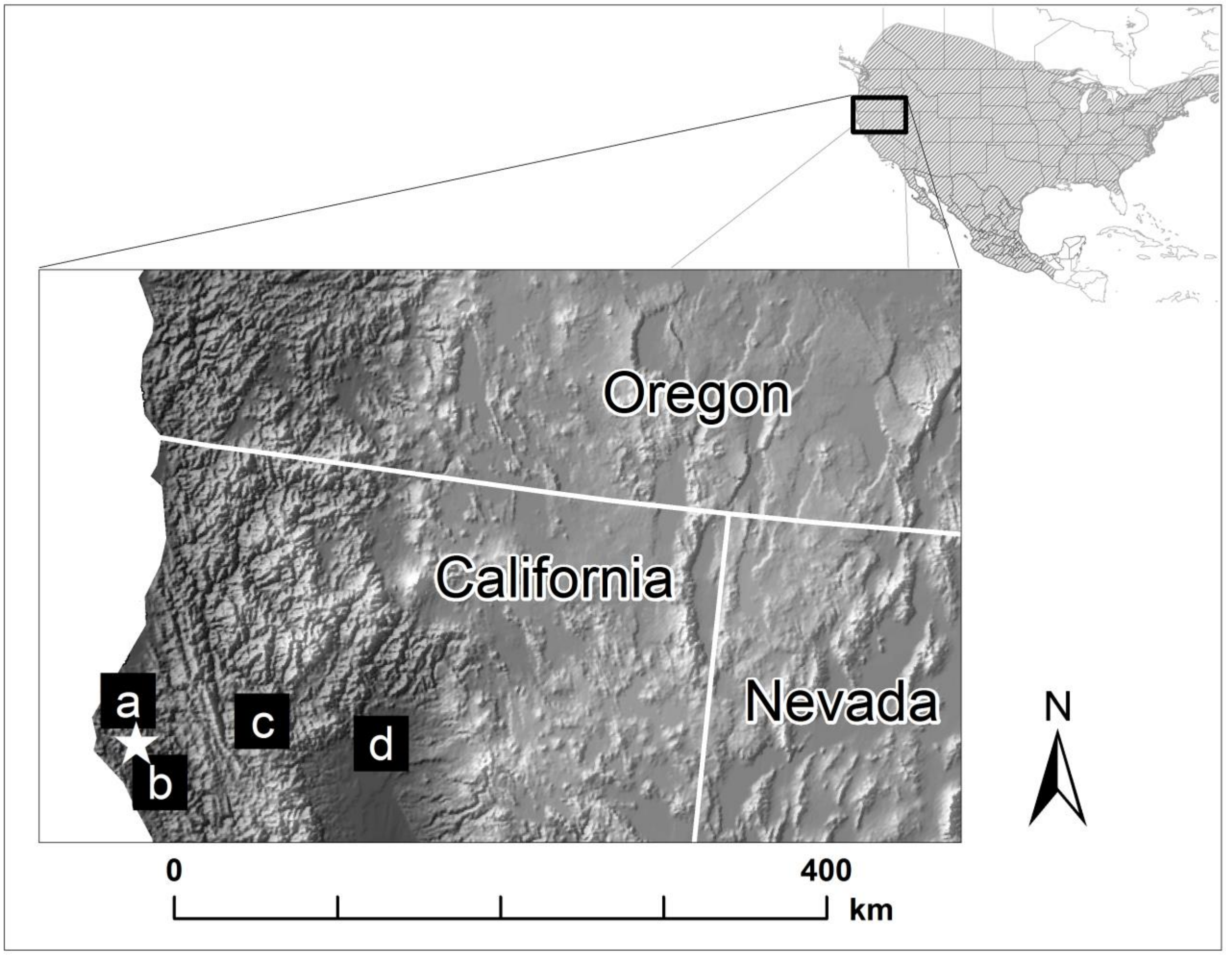

Figure S1. Location where bats were tagged and recaptured is shown with a white star. Weather stations, shown as black squares, were: a) KFOT (latitude 40.552960, longitude -124.133380, elevation 155 m) 25 km NNW, b) ERCC1 (40.138375, -123.823758, 145 m) 28km SSE; c) HYFC1 (40.548514, -123.165133, 709 m) 75km ENE; and d) KRDD (40.514610, -122.297700, 152 m) 146km E of our study area. We acquired archived ambient weather conditions for nearby weather stations from the MesoWest Project (http://mesowest.utah.edu/) to characterize temperature and weather conditions in areas where bats may have been present. Map created usingArcGIS version number 10.3.1 (http://www.esri.com/software/arcgis)**.**

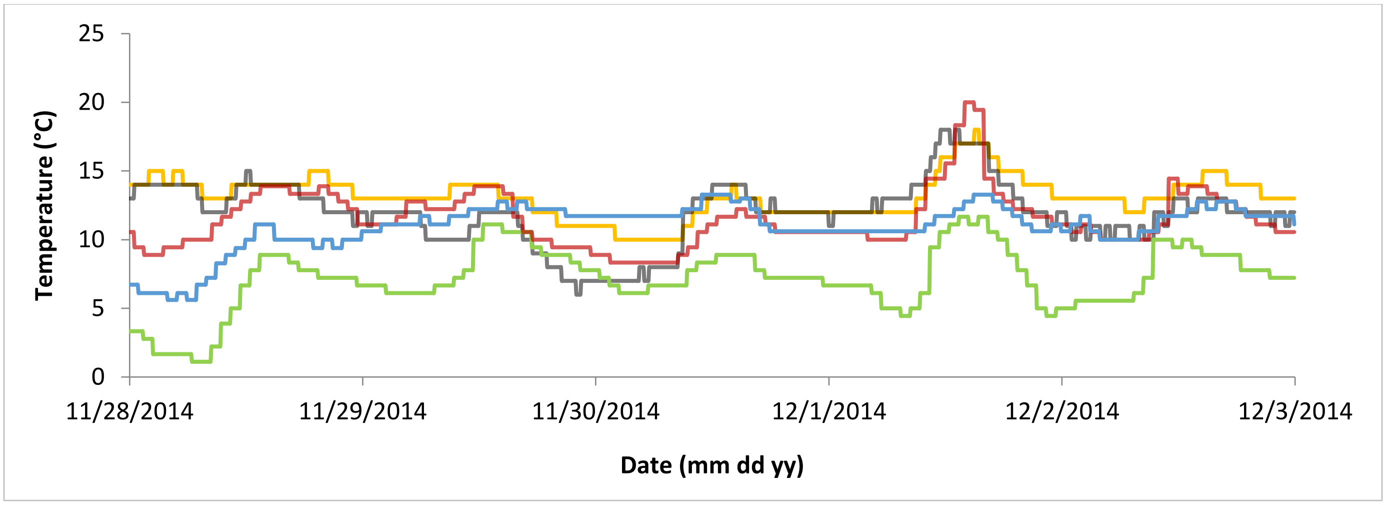

Figure S2. Tag temperature (yellow) compared to temperature at KFOT (gray), ERCC (red), KRDD (blue), and HYFC (green) weather stations. The bat was inactive and torpid during this time period and tag temperature conformed to ambient temperatures. We used this information to conclude that the bat was in the vicinity of where it was captured: 25 km SSE of KFOT station and 28 km NNW of ERCC station.

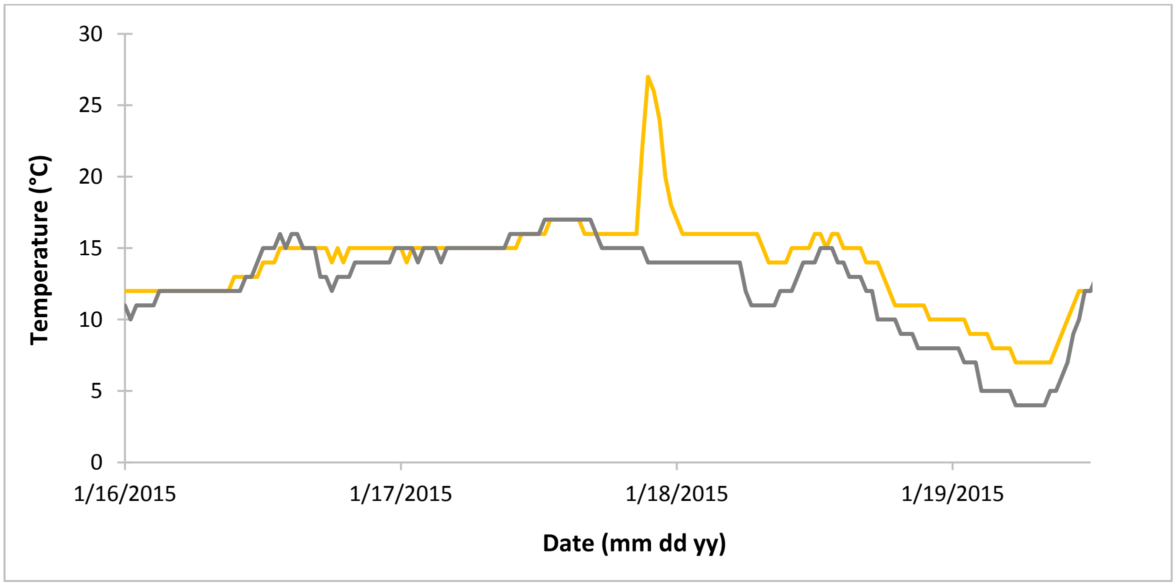

Figure S3. Tag temperature (yellow) compared to ambient temperature at KFOT weather station (gray) during mid-January 2015. Tag temperature rose sharply above ambient around 21:00 on the night of 17 Jan 2015 for about 3 hours. Activity levels associated with flight were not recorded during the arousal. This indicates that bat aroused from torpor without taking flight during this period.
